# Assessing Resources in a Population of Hemodialysis Patients: A New Approach to Improve Quality of Care

**DOI:** 10.1007/s10879-021-09524-5

**Published:** 2021-10-27

**Authors:** Tanja Bellier-Teichmann, Matteo Antonini, Philippe Delmas

**Affiliations:** La Source, School of Nursing, HES-SO University of Applied Sciences and Arts Western Switzerland, Lausanne, Switzerland

**Keywords:** Positive psychology intervention, Resources assessment, AERES, Consensual qualitative research, Wellbeing

## Abstract

Hemodialysis patients constitute a vulnerable population. Their health needs are considerable and they often present psychological symptoms such as depression and anxiety. Empirical studies have demonstrated the efficacy of positive psychology interventions to enhance the well-being of patients and alleviate their depressive symptoms. One such intervention consists in identifying and mobilizing patient resources to activate their recovery. An intervention of the sort was implemented in Switzerland with hemodialysis nurses using AERES, a novel self-assessment instrument. AERES covers 31 domains under three dimensions: personal characteristics/qualities, hobbies/passions, and social/environmental resources. The aim of this qualitative study was to explore hemodialysis nurse perceptions of the use of this instrument. Sixteen hemodialysis nurses were recruited in six hospitals in French-speaking Switzerland and interviewed after delivering the intervention. A consensual qualitative research method was used to analyze the data. Results showed that the resources instrument was easy to administer and beneficial to patients and health professionals. Patient wellbeing became the top priority for the nurses and new interventions centered on patient resources were undertaken. Quality of patient care was improved. Nurses perceived this positive psychology instrument as a means of creating a positive relationship with patients and supporting them emotionally. Assessing the resources of this vulnerable population can provide health professionals with a powerful tool to understand patient intact resources, which can be used to alleviate symptoms and foster wellbeing.

## Introduction

As life expectancy continues to grow and healthcare actions against acute illnesses become more and more effective, Western countries are seeing a sharp rise in the incidence of chronic diseases. According to the 2010 Global Burden of Disease study, chronic kidney disease (CKD) went from 27th on the list of causes of total number of deaths worldwide in 1990 to 18th in 2010. When CKD progresses to its final phase, end-stage renal disease, kidney replacement becomes necessary. However, if this is not possible and in the wait for a transplant, kidney activity must be supported by long-term renal replacement therapy. The most widely used of these is hemodialysis, a procedure that demands an enormous commitment from patients. Numerous symptoms and side-effects are associated with hemodialysis (for a review, see Almutary, Bonner, & Douglas, 2013). The most common of these are fatigue or lack of energy (81% of patients affected), feeling drowsy (75%), and pain (65%). Psychological symptoms such as depression and anxiety are often present and undertreated in this population (Almutary et al., 2013; Rabindranath et al., 2005). These symptoms have a heavy impact on their quality of life (QoL; Mollaoglu, 2004), which is a key indicator of their survival in the short term (Tsai et al., 2010). This situation is common to many chronic diseases and calls for a new approach and new interventions that would be beneficial in terms of improving patient well-being and alleviating psychological symptoms despite the presence of a chronic illness.

Positive psychology interventions (PPIs) are a promising approach to well-being enhancement. PPIs can be defined as treatment methods or intentional activities that aim to cultivate positive feelings, behaviors, or cognitions (Sin & Lyubomirsky, 2009). They are based on (a) cultivating positive subjective experiences, (b) building positive individual traits, or (c) building civic virtue and positive institutions (Meyers, Van Woerkom, & Bakker, 2013). Empirical studies have demonstrated the efficacy of PPIs such as counting your blessings (Emmons and McCullough 2003; Seligman et al., 2005), practicing kindness (Otake et al., 2006), setting personal goals (Green, Oades, & Grant, 2006), expressing gratitude (Seligman et al., 2005; Sheldon & Lyubomirsky, 2006), and identifying and using personal strengths and resources (Seligman et al., 2005). Sin and Lyubomirsky (2009) conducted a meta-analytical review of 51 PPIs with 4266 individuals. Their results showed that PPIs are effective in enhancing well-being and help reduce depressive symptom levels in clinical populations. PPIs may be an effective option for treating different mental disorders, including anxiety disorders (Fava et al., 2005) and depression (Sin & Lyubomirsky, 2009). Furthermore, they can be useful for increasing positive affect, engagement, and life meaning, which are generally diminished when depressive symptoms are present (Forbes & Dahl, 2005; Seligman, Rashid, & Parks, 2006). One type of PPI consists in identifying patient strengths and resources. Patient resources may be defined as a set of elements that protect against problems and promote QoL and wellbeing (Rapp & Goscha, 2012). This approach emphasizes the need for professional healthcare to focus on patient potential. Indeed, assessing resources and positive functioning serves a preventative function against future psychopathology and relapse (Fredrickson, 2001; Watson & Naragon-Gainey, 2010; Wood & Joseph, 2010). Additionally, when resources and symptoms are assessed jointly, patients are more likely to experience this intervention as affirming, empowering and motivating (Saleebey, 2006). Mobilizing patient resources may help patients cope better with stress and, in turn, improve their health status. Specifically, Bellier-Teichmann, Golay and Pomini (2018) showed that performing a strength-based assessment was a therapeutic process that could foster recovery. Indeed, assessing personal resources, strengths and abilities afforded two key benefits. On the one hand, it provided a holistic and more balanced view of patients. On the other, it could serve to support patient recovery. Assessing positive functioning also helps promote wellbeing and enhance creativity in problem solving (Fredrickson, 2001). In sum, patients in general stand to gain from having their strengths and resources assessed alongside their problems and vulnerabilities, and this is perhaps all the more true for patients suffering from chronic diseases. This is why patient resources assessment should be a systematic component of the initial evaluation procedure of any clinical consultation and an ongoing consideration throughout the course of any healthcare treatment.

In the aim of using PPIs in care settings, we have been developing an educational intervention for nurses on an ongoing basis for over a decade. The intervention has been pilot implemented twice over the years, once in Quebec (O’Reilly & Cara, 2011) and later in Switzerland (Delmas et al., 2016). A further component of the intervention was introduced in 2018 (Delmas et al., 2018) to train nurses in the use of an innovative patient resources self-assessment tool called AERES (from French “Auto-Evaluation des RESsources”: resources self-evaluation, see Bellier-Teichmann, Golay, & Pomini 2018). The effects of the latest iteration of the educational intervention were evaluated through a mixed-methods cluster randomized controlled trial (RCT) in a sample of 100 nurses (Delmas et al., 2018).

The purpose of this paper is to report part of the qualitative results of this research. In particular, it focuses on a qualitative exploration of how hemodialysis nurses who received the educational intervention perceived their use of AERES, the patient resources self-assessment tool. As our tool was developed in the field of psychiatry, we are interested in the users’ experience in other healthcare contexts to further develop this instrument.

## Methods

### Design

The design chosen for the study was Consensual Qualitative Research (Hill 2005, 2012, 2015), a data-driven method of analysis based on team consensus. It entails the systematic evaluation of thematic representativeness across multiple cases. This research was part of a broader mixed-methods randomized control trial of the educational intervention (Delmas et al., 2018). Fifty hemodialysis nurses in the experimental group received the intervention and then 16 of them participated in a series of semi-structured interviews that served to qualitatively explore their perceptions of the EI. In this paper, we focus on their perceptions of the use of the patient resources self-assessment tool.

### Educational Intervention

Rooted in a humanistic caring approach, the educational intervention consisted of four 3.5-h sessions delivered at the rate of one per week over 4 weeks (Delmas et al., 2018). To optimize the learning experience, groups were limited to no more than five nurses. Each session was structured as follows. For starters, a focusing exercise was proposed to sharpen participant concentration and to clearly delineate time dedicated to the educational intervention from time dedicated to other activities. Then, participants engaged in the main part of the EI. This activity varied from session to session. The first session introduced the core concepts of Watson’s human caring and included an open discussion on the situation of hemodialysis patients (Watson, 2008, 2012). The second session introduces the AERES resources self-assessment tool. Nurses were trained in the use of the tool and were asked to try it out with their patients. AERES was used as both an exercise, to revise the concepts and test the capabilities assimilated so far, and as a small intervention to understand how to use the tool in a real-life context. This session was modified from the original structure of the educational intervention to introduce AERES and focus specifically on the PPI. During the third session, nurses shared their experiences regarding the PPI tool and discussed how patients reacted to it. In the fourth session, a simulation was organized to review the concepts discussed in the previous sessions and to put them into practice. The last session ended with an open discussion to collect feedback from participants and instructors.

### AERES

AERES is a novel instrument used by patients to self-assess their personal resources (Bellier-Teichmann et al., 2018). It was developed with three objectives in mind. First, it had to serve to describe all of a patient’s main internal and external resources as defined in the scientific literature. Second, it had to be easy and fast to use even with patients with cognitive or language limitations. Third, it had to provide a general profile of a patient’s resources to be used to plan clinical or psychosocial interventions to enhance and amplify their resources. The instrument that we used was composed of 31 cards, each of which depicted a separate resource. The resources fell into three sets: (1) personal characteristics/qualities, (2) hobbies/passions, and (3) social/environmental resources. A blank card completed the deck to afford the opportunity to add further resources and to tailor the instrument to each patient. Self-administration of AERES was a three-step process. First, patients had to indicate whether a given resource was present or absent. Second, they had to rate how much the resources present contributed to their recovery based on the following scale: “not at all”, “a little”, “moderately”, and “strongly”. Third, patients were asked whether they would like to develop new resources or potentiate existing ones. AERES was successfully pre-tested in two small-sample pilot studies by Bellier-Teichmann and Pomini (2015) and Bellier-Teichmann, Fusi and Pomini (2017) to determine the feasibility and acceptability of the instrument with patients. In a large-scale validation study with 213 participants, the instrument demonstrated robust psychometric properties (Bellier-Teichmann et al., 2018).

### Sample and Recruitment

Our sample comprised 16 nurses drawn from all six hemodialysis units included in the experimental sample. The hemodialysis units were all situated in French-speaking Switzerland. Given the heterogeneity of staff across units, we weighted the number of participants based on hemodialysis unit sizes. Accordingly, larger hemodialysis units provided more participants. Nurses had to meet two inclusion criteria: (1) had to have been working in their unit for at least 6 months, and (2) had to consent to participate in study. Nurses who intended to leave their unit in the next three months were excluded. A convenience sample was selected on this basis. According to Hill’s guidelines (2012, 2015), at least eight participants were needed for Consensual Qualitative Research and at least 16 if researchers wished to consider rare categories.

### Sociodemographic Characteristics

Of the 16 nurses, 5 were from the Jura Hospital in Delémont et Porrentruy, 2 from the Valais Hospital in Martigny, 1 from Morges Hospital, 3 from the Broye Intercantonal Hospital in Payerne, 2 from the Riviera-Chablais Hospital in Monthey, and 3 from the North Vaud Hospital in Yverdon. The sample included 14 women and 2 men, which reflected the gender split among nurses in the hemodialysis units in French-speaking Switzerland (Addor et al., 2016). Their mean age was 44 years (SD = 8.8) and their mean years of experience working in hemodialysis was 11 (SD = 6.8). On average, they worked 77.8% (SD = 17.2) of a full-time position.

### Data Collection

Semi-structured interviews were conducted with each of the participating nurses by a researcher with expertise in qualitative interviews. An interview guide was developed by the research team for the purposes of the study. Interviews took place from May to October 2018, ten weeks after the end of the educational intervention at each hemodialysis unit, at the nurses’ workplace and lasted about 60 min. All interviews were recorded, transcribed and anonymized using an alphanumeric code by the researcher in charge of the qualitative component of the study. Quality control was exercised by the research coordinator, who listened to parts of the recordings against the transcripts produced.

### Ethical Considerations

The research was approved by the Vaud Research Ethics Committee (CER-VD), which was the body with regional jurisdiction (N 2017-00946). The nurses at each hemodialysis unit were met beforehand to probe their interest in participating in the study. Then, interested parties received an information letter laying out the objectives and methods of the study and a consent form. Nurses were given a week, or more if needed, to weigh the information and return the consent form. They were advised that they would be free to withdraw from the study at any time without justification or consequences. All of the data collected were saved on a secure institutional server or stored in secure physical archives. Only authorized persons had access to the data.

### Data Analysis

Interviews were analyzed using the Consensual Qualitative Research (Hill et al., 2005, 2012, 2015), which combines a constructivist approach (emphasis on individual expression) with a postpositivist approach (data synthesis through consensus seeking). It holds several advantages over other qualitative research techniques: (1) use of open-ended questions in semi-structured interviews, (2) centrality of research team consensus in data analysis, (3) use of one or more auditors to cross-check data, and (4) cross-analysis of data at “domain” level.

In practice, the Consensual Qualitative Research method comprises four interconnected steps. First, interviews are conducted and transcribed. Second, domains are defined. In our case, domains were developed based on the results of previous studies and from what emerged from interviews. As per the guidelines of the Consensual Qualitative Research (Hill, 2015), five interviews were selected consensually and in accordance with the criteria of content clarity and heterogeneity. These were used to define the coding scheme used to analyze the other interviews and to create our list of domains consensually. It is at this step that domains and general semantic categories are defined. Third, core ideas are identified. Every interview was segmented to identify its core ideas. As per the Consensual Qualitative Research (Hill 2012), each segment was reduced to a short topic sentence. The terms and expressions used by the participants were retained as much as possible. The core idea should express the essence of each segment of the transcript. Fourth, domains and core ideas are validated for each participant by way of consensus. The researchers discussed each core idea to crystallize its final wording and to assign it to a domain consensually. Consensus seeking is central to Consensual Qualitative Research. Bringing multiple viewpoints to bear when analyzing interviews makes it easier to deal with their complexity and specificity. An external auditor then reviewed the results for consistency between raw data and final core ideas (Hill et al., 2005). Finally, representativeness was determined by calculating the frequency of occurrence of each domain and category.

### Research Team

Our research team included an expert in qualitative interviews with a background in life-course sociology. Vanessa Brandalesi (La Source, School of Nursing) conducted all the interviews and transcribed them. Tanja Bellier-Teichmann, the team coordinator, had a PhD in clinical psychology. Specialized in qualitative research methods and specifically in Consensual Qualitative Research, she coordinated data analysis in conjunction with Philippe Delmas, the principal investigator of the overarching RCT. This person held a PhD in nursing and had extensive experience in the study of hemodialysis patients and the creation of humanistic caring interventions, not to mention extensive expertise in qualitative methods. Matteo Antonini held a PhD in social sciences and specialized in life-course studies. He participated in analyzing the interviews.

Finally, Delphine Roulet Schwab (La Source, School of Nursing) was an external researcher with a PhD in psychology, a specialization in gerontology and clinical psycho-sociology, and expertise in qualitative research. The external auditor was involved in the project at each step of the analysis to provide feedback to the research team.

## Results

Seven domains emerged from the interviews. Four were defined on the basis of the content of the first five interviews and in accordance with the content clarity and heterogeneity criteria. The other three were identified via analysis of the last 11 interviews. The domains were defined as follows: (i) transformation of nursing clinical practice, (ii) transformation of team spirit in care team, (iii) effects of the educational intervention on nurses, (iv) effects of the educational intervention on patients as perceived by nurses, (v) educational intervention features, (vi) contextual barriers, and (vii) other elements. This last domain was added in accordance with Consensual Qualitative Research guidelines (Hill 2012). Table 1 provides an overview on all the domains, categories and subcategories generated during the analysis. Of these seven domains, two included elements that were relevant to the purpose of this study, i.e. they are directly linked to nurses’ perceptions of the patient resources self-assessment tool: “transformation of nursing clinical practice” and “educational intervention features”.

**Table 1:** General analysis grid following Consensual Qualitative Research guidelines (Hill et al., 2005): domains, categories and subcategories to emerge from the study’s qualitative analysis

Domains	Categories	Subcategories
Transformation of nursing clinical practice	Humanistic practice was strengthened	Patient-centered practice was strengthened
		Importance of relational care was underscored
		Listening attitude was strengthened
		Patient wellbeing became nurses’ top priority
		NPR was improved
	New practices emerged	New interventions were undertaken or planned
		New perspective and common language emerged
		Self-reflection and reflection on personal practice emerged
	Some limitations emerged	No change in practice was perceived following educational intervention
Transformation of team spirit in care team	Quantity and quality of interactions between nurses were enhanced	Nurses interacted more with patients about their lived experiences
		Support and solidarity between nurses were strengthened
		Climate at work became more pleasant
	Some limitations emerged	No change in team spirit was noted
Educational intervention effects on nurses	Quality of work life was improved	Nurse wellbeing at work improved
		Negative emotions at work diminished
		Situations with patients considered difficult were managed with greater serenity
	Perception of self and profession was changed	Nurses perceived value and legitimacy of their profession more
		Self-awareness was raised
Effects on patients perceived by nurses	Quality of life of patients was improved	Patients shared their lived experiences more with nurses
		Patients perceived an improvement in treatment from nurses
		A positive emotional state promoted among patients
EI features	Content and pedagogical approach fostered an enhancement of practice	Content and pedagogical approach of training were beneficial
		Exchanges between instructors and participating nurses were beneficial
		Content of training was appreciated
		Training warrants wider dissemination
	Tool provided added value	Tool perceived as beneficial
		Tool promoted relational closeness with patients
	Tool has limitations	Recommendations made on how to improve training
Contextual barriers	Work context constitutes a barrier to application of a humanistic practice	A crisis situation was identified in the facility
		Management not centered on caring is source of resistance against training
		How work is organized constitutes a barrier to applying humanistic care
Other	Several characteristics of team prior to training named	Presence of problems and dehumanizing practices were identified within the team
		Positive dimensions were identified in team
		Presence of neutral elements and some questions were identified following training

### Domain: Transformation of Nursing Clinical Practice

The first domain comprised three categories: (i) humanistic practice was strengthened, (ii) new practices emerged, and (iii) some limitations emerged. The first two were directly connected to the identification of patient resources and the use of AERES. Limitations are not discussed as they include no element directly link to AERES, but rather describe a general lack of change in nurses’ everyday work described as already rooted in a humanist perspective.

#### Category: Humanistic Practice was Strengthened

All 16 nurses indicated that the educational intervention contributed to strengthen humanistic practice. This category broke down into five subcategories: (i) patient-centered practice was strengthened, (ii) importance of relational care was underscored, (iii) listening attitude was strengthened, (iv) patient wellbeing became nurse top priority, and (v) nurse–patient relationship (NPR) was improved.

The first subcategory was relevant to the purpose of our study. Every nurse stated that patient-centered practice had been strengthened thanks to the EI. This subcategory comprised four core ideas. They described how nurse practices became more humanistic by focusing more on the potential and uniqueness of each patient. This was made possible through use of AERES. Nurses emphasized four points. First, (i) patient wellbeing became their top priority. Second, it awakened (ii) a new resolve to bring patients positivity, peace and benevolence. Third, it helped them (iii) deepen their analysis of and ascribe greater importance to patient resources as a means of improving patient wellbeing. Fourth, these changes led, in particular, to (iv) better management of patient aggression: “Yeah, I was amazed to get results with this tool; […] he can be aggressive with his caregivers. But with this tool, though, within five minutes of using it, the patient just opened up and it pleased him and I saw him smile and laugh for the first time in months and months” (P01).

#### Category: New Practices Emerged

Every nurse also indicated that new practices directly linked to the identification and amplification of patient resources emerged thanks to the EI. This category broke down into three subcategories: (i) new positive interventions were undertaken or planned, (ii) nurses looked at patients differently and acquired a new common language, and (iii) nurses reflected more on their identity and practice.

The first subcategory was directly connected to the development of patient resources and the use of AERES. This was a typical subcategory, as it was mentioned by 11 of the 16 nurses. After the EI, some nurses undertook new projects to further develop their practice. This was a significant and unexpected effect of the EI, even though it was limited to a few hemodialysis units. For example, several nurses began reporting on their patients’ emotional status and highlighted their resources at weekly meetings. This procedure allowed continuous monitoring of both the physical and emotional status of patients in a holistic manner.

### Domain: Educational Intervention Features

This second domain broke down into three categories: (i) the educational intervention raised nurses’ clinical practice through content and pedagogical approach, (ii) AERES provided added value, and (iii) the educational intervention presented some limitations. Again, limitations are not discussed as they include no element directly link to AERES.

#### Category: Educational Intervention Raised Nurses’ Clinical Practice

This category comprised two subcategories: (i) tool (AERES) was perceived as beneficial, and (ii) tool (AERES) promoted relational closeness with patients.

Twelve core ideas specified why nurses perceived AERES as beneficial. These covered how their perspective changed as a result of using the instrument and the role the instrument played during the EI.

Regarding the change in perspective, nurses stressed that using AERES reinforced the idea that (i) trying to find solutions for patients constrained the nurse’s role whereas finding solutions with patients facilitated problem solving. Moreover, (ii) identifying patient resources was a starting point to support patient wellbeing and (iii) using AERES for this purpose could improve the process of care. This was possible because AERES (iv) provided access to the whole patient and it could (v) lead nurses to discover hidden aspects of themselves.

Given these results, nurses (vi) appreciated the role played by the instrument in the EI. They found the instrument to be (vii) beneficial on a personal level, (viii) interesting, (ix) a turning point in their education, and (x) pleasant to use. They also felt (xi) a sense of accomplishment and satisfaction when they used it with patients. Finally, nurses reported (xii) better management of patient aggression after AERES was incorporated in their practice.

The central role of patient resources was clearly perceived by nurses. As one nurse put it:

I didn’t use 36 tools, just one. To help mobilize resources. And for the patients, those who are more difficult, let’s say, at the relational level, well it helped a lot (P01).

Finally, this strategy led to a holistic view of the patient: “Yeah, I found that part of the training really interesting, the exercise on resources…it opened doors to the whole patient, I find” (P05).

This shift in paradigm also had effects on how nurses perceived themselves: “I really appreciated that exercise a lot […]. I really did, because, let me tell you, it made me discover things about myself” (P09).

The second subcategory comprised six core ideas that specified how AERES changed the NPR. Nurses stressed that, by using the tool, they improved both (i) relational closeness with patients and (ii) trust. Moreover, (iii) it helped resolve tense situations with patients and (iv) it contributed to a positional shift in how nurses looked at patients. This was possible because, after using AERES, (v) patients were more open to talking and (vi) it was easier to identify the resources that might solve problem situations. One nurse said this: “When I started with the questionnaire, [..] I understood then that he was angry not at me, but at his condition. When I asked him about his resources, he opened up and he began to talk, and I heard him talk like never before. And just like that, the tension between us broke” (P02).

Openness was often accompanied by trust: “I didn’t know how to approach him again, to re-establish a connection, to gain his trust, and so the discussion that we had about resources changed everything” (P06).

These elements greatly improved the quality of the NPR: “I believe that a different relationship came about after identifying the patients’ resources” (P03).

## Discussion

The purpose of our study was to explore how hemodialysis nurses perceived the use of a patient resources self-assessment tool. The use of AERES with patients was framed in a larger educational intervention, addressed to nurses, to improve the quality of the nurse–patient relationship. Two domains were identified that related to their perception of using AERES. The first translated, in practical terms, into a strengthening of humanistic practice centered on patient resources and the emergence of new positive practices. The second evidenced that the resources assessment tool provided added value. This category broke down into two subcategories: tool was perceived as beneficial and tool promoted relational closeness with patients.

### Strengthening Nurses’ Humanistic Practice

Regarding strengthening of humanistic practice centered on patient resources, nurses underscored that patient wellbeing became the top priority of care. They expressed the resolve to present patients with a positive outlook and take their resources into account more. As reported in the literature, hemodialysis nurses often focus on managing the technical aspect of HD care exclusively (Bennett, 2011; Bevan, 1998). Moreover, nurses also indicated that they used to be focused essentially on managing patient symptoms inherent to hemodialysis. After receiving the educational intervention and using AERES, they acquired a heightened awareness of the importance of identifying and supporting the internal and external resources of patients. This effort of focusing on resources and health protective factors constitutes a first step towards preserving and enhancing patient wellbeing. This is a key element that holds tremendous potential to promote mental health in this population (Delmas et al., 2018). AERES also supports a salutogenic approach (Antonovsky, 1987), which entails a change in how nurses look at hemodialysis patients and a new way of managing them. This is a central reason for introducing this instrument in a nurse EI. Indeed, though nursing education is based on a salutogenic approach, clinical practice continues to be dominated by the positivist medical model. The use of instruments such as AERES allows nurses to return to their essence by supporting the QoL of hemodialysis patients.

In terms of change in clinical practice, one nurse stated that identifying patient resources made it possible to better manage aggression in some patients. According to this nurse, assessing a patient’s strengths can help break the tension or aggression sometimes found in hemodialysis patients, which often stems from the complexities and challenges that being on hemodialysis can entail. According to Epstein (2000), identifying a patient’s personal resources improves their capacity to deal effectively with stress or adversity and fosters their personal and social development. Being aware of one’s personal resources is believed to contribute to diminish stress and to increase positive emotions and wellbeing (Murdoch et al., 2020; Wood & Tarrier, 2010). Furthermore, the exercise of identifying a patient’s resources reportedly provides a first dose of relief from psychological suffering, such as anxiety or depression (Mehran & Guelfi, 2002), which are recurring symptoms among hemodialysis patients. According to Graybeal (2001), the mere fact of conducting an assessment of a patient’s resources is, in and of itself, therapeutic. This sort of positive effect produced by the identification of personal resources and demonstrated through empirical research might explain why, according to the nurses in our study, the tool contributed to diminish aggression or tension in hemodialysis patients.

### The Emergence of New Practices

Regarding our second finding, the educational intervention opened up new possibilities for nurses, which might explain the emergence and creation of interventions dealing directly with the identification and potentiation of patient resources. One nurse expressed the wish to create an electronic file to help trace the life history of patients and take inventory of personal resources. The educational intervention and particularly the use of AERES allowed nurses to mobilize. They took the initiative to propose customized follow-up for hemodialysis patients by creating an electronic file to help trace and highlight their resources. The fact that nurses embraced the instrument very quickly attests to its user-friendliness and to a specific interest in this PPI approach. It is not unreasonable to conclude, then, that AERES shows good acceptability, relevance, and clinical usefulness. Changing care practices to integrate PPI tools is no easy feat and often runs up against time considerations and the reality on the field. In our case, the tool was integrated in practice as a means of getting to know the other person and gaining a better understanding of individual hemodialysis patients in their entirety. This new use of a resources assessment tool was perceived as a time saver given that the quality of both the NPR and care was improved. This type of assessment in fact enriches the patient’s medical history and broadens the array of possible PPIs that nurses can deliver to support the wellbeing of hemodialysis patients instead of merely seeking to reduce their physical symptoms and impairments. AERES thus constitutes not only a theoretical tool but also a practical one in that it is rooted in a validated, standardized and systematized methodology that expands the toolkit of health professionals in their positive clinical practice. This collection of data contributes here more specifically to broaden the scope of nursing strengths-based care practice and to valorize the true role of nurses, the role that is their preserve, a role that is often ill known, underestimated and undervalued. The literature shows in fact that this type of assessment centered on patient strengths allows building new care possibilities by exploiting these strengths (Mehran & Guelfi, 2002). Furthermore, identifying the patient’s positive and functional aspects may provide invaluable information on how to foster recovery (Kuyken, Padesky, & Dudley, 2011; Rapp & Goscha, 2012; Rashid & Ostermann, 2009). Recovery has been defined in psychiatry as the possibility of leading a rewarding life in society, which includes possessing a positive identity based on hope and self-determination despite limitations associated with extant symptoms (Bonsack & Favrod, 2013; Slade, 2010). To promote recovery, it is necessary, then, to be able to identify and reinforce patient resources despite persistent and at times invasive symptoms (Repper & Perkins, 2003). Recovery and wellbeing do not boil down to the reduction of physical symptoms alone; they also entail the presence of positive emotional and cognitive states (Joseph & Wood, 2010; Slade, 2010). However, though the nursing profession has often championed the salutogenic approach, such as exemplified by strengths-based nursing care (Gottlieb, 2013), this approach has often remained a purely theoretical model to be promoted without operational tools to realize it. AERES operationalizes the model and supports the visibility of a salutogenic management of hemodialysis patients. Thus, identifying resources with an instrument fosters a holistic view of patients and allows identifying the protective factors that promote QoL despite the presence of symptoms.

### AERES Was Perceived as Beneficial

Our third finding evidences the benefits of the resources assessment tool perceived by nurses both for themselves and for patients. First, nurses reported that they derived a sense of pleasure from using the tool. They indicated that the tool was fun and enjoyable to use and that they perceived it as a game. Second, they also described a sense of accomplishment and meaning with respect to the perceived benefits of this tool in their clinical practice. According to the nurses, the self-assessment tool improved the quality of patient management by opening doors on the whole patient. It thus fosters a shift in perspective and allows strengthening an approach centered on patient strengths to support their wellbeing. These results are consistent with hypotheses derived from the stress-vulnerability model to the effect that resources provide protection against the deleterious effects resulting from the interaction between individual vulnerabilities and environmental or life-event stressors (Leclerc, Lesage, & Ricard, 1997).

### AERES Promoted Relational Closeness with Patients

Our fourth finding shows that using AERES promotes relational closeness with patients. The nurses underscored that identifying resources fostered a relationship of trust with patients. It also led to a shift in stance among patients, that is, they confided more easily in the nurses caring for them. This result might be explained by the fact that when patients feel valorized in terms of their strengths, they are more motivated to identify and talk about their problems and troublesome emotional states (Flückiger et al., 2009; Grawe, 2006; Grosse Holtforth et al., 2007). It has been proposed that this dual approach involving the simultaneous treatment of symptoms and the identification of strengths sends the message to patients that caregivers recognize them as individuals with competencies and not just impairments and symptoms (Saleebey, 2006). Moreover, when strengths and risks are assessed at the same time, patients experience the assessment as more supportive, motivating and conducive to a sense of empowerment (Saleebey, 2006). Consequently, it seems essential to support this dual approach in all care processes and to apply it from the outset when taking the patient’s medical history. This approach also allows viewing patients in a more holistic manner.

Another aspect of AERES no doubt promoted relational closeness and strengthened the relationship of trust between caregivers and patients, namely, the use of self-assessment. The tool’s self-assessment approach allows focusing on the patient and taking account of their views and perspective. In this regard, systematic literature reviews have shown that when caregivers and patients collaborate to identify problems and needs, define objectives, and make decisions jointly, patients are more satisfied, they trust their caregivers more, and their symptoms diminish more rapidly (Rao et al., 2007; Stewart et al., 2000). This new approach to care using self-assessment allows patients to change roles. Instead of being a passive object of treatment, they become active subjects and, therefore, agents within care services. Taking account of their subjective perception fosters their motivational resource and their engagement. Empowering patients results in more effective care. This is why proposing a tool that allows obtaining the patient’s viewpoint on their resources while limiting interference from health professionals can be beneficial. Taking into consideration the patient’s perception and perspective is no doubt what fostered the greater relational closeness perceived by nurses. Also, the nurses indicated that, thanks to this tool, they had the impression of discovering not a patient but a person with a life history and life experiences, which included kidney failure and hemodialysis but also strengths, resources and potentials. It is in this sense that the instrument for self-assessing resources lays the groundworks for a strengths-based care practice. It facilitates the work of nurses and fosters the development of strengths-based attitudes and behaviors by providing concrete support.

### Limitations

This study explored how nurses perceive the use of AERES in their clinical practice. In order to measure the impact of this type of instrument more in depth, it would be interesting to explore how hemodialysis patients perceive identifying their resources. Furthermore, it would be useful to complement our findings by including a measure of how identifying personal resources impacts patient wellbeing and QoL over the long term.

Also, the lack of quantitative data, the limited size and the homogeneity of our sample (all the nursing working in HD units) limit the extent of our results.

## Conclusion

The results of our study indicate that nurses perceive identifying patient resources as beneficial. They indicated that AERES was easy to administer and met with appreciation. This is the first study to explore the use by hemodialysis nurses of a resources self-assessment instrument in a population of hemodialysis patients to examine their resources as a whole. Using such an assessment in clinical practice may counterbalance the pathogenic approach to care centered primarily on patient symptoms. Indeed, under the classic view of medicine and psychology, the prime objective of the care team is to reduce patient symptoms. Once identified, symptoms become the target of pharmacological treatments or psychotherapy and the effectiveness of the treatment is measured by their impact on symptoms. As it happens, this medical approach tends to objectify patients, who are considered as passive people without freedom of choice and responsibility (Deegan et al. 2008). The benefits of PPIs such as identifying resources have been documented in numerous empirical studies (Bellier-Teichmann et al., 2018; Fredrickson, 2001; Saleebey, 2006; Watson & Naragon-Gainey, 2010; Wood & Joseph, 2010). Moreover, a high number of studies have fostered a change in care practices allowing patients to regain control over their life, decide about the treatments and care they receive, and promote their recovery by relying on their talents and strengths (Deegan et al. 2008; Slade, 2017; Bellier-Teichmann et al., 2018; Bellier-Teichmann & Pomini, 2018). In this regard, AERES falls within a shift in perspective geared to helping patients and care teams engage in a shared care approach centered on resources. This tool challenges existing care practices by way of its methodology, its focus on the patient, and its assessment not of patient symptoms but of patient resources. We are currently witnessing a boom in chronic diseases that requires using new approaches and tools involving strengths-based care practices. This includes implementing new tools that facilitate focusing on patient resources and recovery. Instruments such as AERES need to be known better by health services and used more widely, particularly with patients living with chronic conditions.

In sum, AERES may be a positive new addition to the existing battery of measures used with hemodialysis patients, who frequently suffer from many different physical and psychological symptoms, including anxiety and depression (Delmas et al., 2018). Consequently, using this instrument in routine care may provide nurses with information essential to a comprehensive assessment and understanding of the role of resources and their impact on patient recovery. From this point of view, the self-assessment of resources constitutes a motivational process for patients. It can be repeated regularly in order to verify the fit between objectives, the situation and the actual needs of patients. This type of instrument, which is both practical and adaptable to the specific needs of patient populations, remains indispensable to meeting the challenges of a salutogenic approach to care and bringing about a paradigm shift through concrete means. It is hoped that AERES will be integrated into assessments that will lead to better outcomes for patients and foster their recovery, their QoL, and their wellbeing.
